# Pyruvate dehydrogenase kinase regulates hepatitis C virus replication

**DOI:** 10.1038/srep30846

**Published:** 2016-07-29

**Authors:** Gwon-Soo Jung, Jae-Han Jeon, Yeon-Kyung Choi, Se Young Jang, Soo Young Park, Sung-Woo Kim, Jun-Kyu Byun, Mi-Kyung Kim, Sungwoo Lee, Eui-Cheol Shin, In-Kyu Lee, Yu Na Kang, Keun-Gyu Park

**Affiliations:** 1 Department of Internal Medicine, Kyungpook National University School of Medicine, Daegu, Republic of Korea; 2 Leading-edge Research Center for Diabetes and Metabolic Disease, Kyungpook National University Hospital, Daegu, Republic of Korea; 3 Department of Internal Medicine, Keimyung University School of Medicine, Daegu, Republic of Korea; 4 New Drug Development Center, Daegu-Gyeongbuk Medical Innovation Foundation, Daegu, Republic of Korea; 5 Laboratory of Immunology and Infectious Diseases, Graduate School of Medical Science and Engineering, KAIST, Daejeon, Republic of Korea; 6 Department of Pathology, Keimyung University School of Medicine, Daegu, Republic of Korea

## Abstract

During replication, hepatitis C virus (HCV) utilizes macromolecules produced by its host cell. This process requires host cellular metabolic reprogramming to favor elevated levels of aerobic glycolysis. Therefore, we evaluated whether pyruvate dehydrogenase kinase (PDK), a mitochondrial enzyme that promotes aerobic glycolysis, can regulate HCV replication. Levels of c-Myc, hypoxia-inducible factor-1α (HIF-1α), PDK1, PDK3, glucokinase, and serine biosynthetic enzymes were compared between HCV-infected and uninfected human liver and Huh-7.5 cells infected with or without HCV. Protein and mRNA expression of c-Myc, HIF-1α, and glycolytic enzymes were significantly higher in HCV-infected human liver and hepatocytes than in uninfected controls. This increase was accompanied by upregulation of serine biosynthetic enzymes, suggesting cellular metabolism was altered toward facilitated nucleotide synthesis essential for HCV replication. JQ1, a c-Myc inhibitor, and dichloroacetate (DCA), a PDK inhibitor, decreased the expression of glycolytic and serine synthetic enzymes in HCV-infected hepatocytes, resulting in suppressed viral replication. Furthermore, when co-administered with IFN-α or ribavirin, DCA further inhibited viral replication. In summary, HCV reprograms host cell metabolism to favor glycolysis and serine biosynthesis; this is mediated, at least in part, by increased PDK activity, which provides a surplus of nucleotide precursors. Therefore, blocking PDK activity might have therapeutic benefits against HCV replication.

At least 185 million people around the world are infected by hepatitis C virus (HCV)^1^,^2^. Although complications of HCV infection, such as cirrhosis and hepatocellular carcinoma (HCC), develop decades after hepatocellular injury, these complications seriously affect mortality; therefore, optimal and timely management of chronic hepatitis C is necessary^3^. Current standard treatment of hepatitis C consists of the nucleoside analog ribavirin, which blocks guanine nucleotide synthesis, in combination with PEGylated interferon (IFN)-α, which activates the IFN-mediated antiviral response^4^. However, inefficient achievement of sustained virological response has prompted researchers to search for novel therapies. Recently approved antiviral agents include sofosbuvir, simeprevir, and daclatasvir, but the high costs of these drugs has limited their applications in clinical practice^5^,^6^.

Recently accumulated evidence suggests that reprogramming tumor metabolism using glycolytic enzymes represents an effective anticancer strategy^7^,^8^,^9^. In this context, pyruvate dehydrogenase kinase (PDK) is a promising target for cancer metabolic therapy^7^,^10^,^11^,^12^,^13^. PDK phosphorylates pyruvate dehydrogenase (PDH) and inhibits its activity, thereby inhibiting the entry of pyruvate into the TCA cycle^14^. By decreasing the oxidation of glucose, elevated PDK activity in tumor cells provides precursors for macromolecular biosynthesis, such as amino acids and nucleotides^10^,^15^. During aerobic glycolysis (also called the Warburg effect), glycerate 3-phosphate generated from glucose is converted into serine by three consecutive enzymatic cascades; phosphoglycerate dehydrogenase (PHGDH), phosphoserine aminotransferase 1 (PSAT-1), and phosphoserine phosphatase (PSPH)^16^,^17^. Serine hydroxymethyltranferase converts serine into glycine, an amino acid that plays a key role in the biosynthesis of proteins, purines, and glutathiones, as well as in DNA and histone methylation^16^,^17^.

Mounting evidence suggests that metabolic changes that favor aerobic glycolysis and serine/glycine biosynthesis also occur in virus-infected cells; in other words, rapidly replicating viruses modify the metabolism of infected cells in a way that resembles the alterations in rapidly proliferating cancer cells^18^. For example, human cytomegalovirus (HCMV), herpes simplex virus (HSV), human immunodeficiency virus (HIV), and Mayaro virus increase glycolytic flux and reprogram cellular central carbon metabolism to enhance viral replication^19^,^20^,^21^,^22^,^23^. HCV is no exception: the activity of the key glycolytic enzyme hexokinase (HK) is increased by its interaction with the HCV non-structural protein NS5A^24^. Furthermore, HCV infection induces changes that favor glycolytic activity^25^, and expression of PSPH and PSAT-1 is considerably increased in HCV-infected cells than in HCV-uninfected cells^26^. Given that modulation of PDK activity can determine the metabolic balance between oxidative phosphorylation and aerobic glycolysis within a cell^15^, and that serine is derived from the early glycolytic intermediate 3-phosphoglycerate, we reasoned that inhibiting PDK activity would disturb serine/glycine synthesis, thereby inhibiting HCV replication. However, it is unclear whether blocking glycolysis by modulating PDK will inhibit HCV replication, as it does for cancer cells.

In this study, we show that the PDK inhibitor dichloroacetate (DCA) shifts glucose metabolism away from aerobic glycolysis and subsequently inhibits the serine biosynthetic pathway in HCV-infected hepatocytes, thereby blocking HCV replication.

## Results

### Enzymes involved in glycolysis and serine biosynthesis are upregulated in HCV-infected human liver

To evaluate the expression of glycolytic enzymes in HCV-infected human liver, we compared immunohistochemical staining (IHC) in liver tissue obtained from 14 chronic hepatitis C patients and 14 HCV-uninfected controls. The baseline characteristics of the patients are summarized in Table 1. In light of clinical and experimental data showing that patients with HCV have a higher risk of insulin resistance and type 2 diabetes^27^,^28^, we compared clinical metabolic parameters between the two groups. Data derived from 28 HCV-infected human liver samples (14 data sets from IHC and 14 from mRNA analysis) and from 28 HCV-uninfected human liver (14 data sets from IHC and 14 from mRNA analysis) were analyzed (Table 1). The prevalence of diabetes (32.1% in the infected group *vs.* 25.0% in the uninfected group; *p* = 0.768) and hypertension (25.0% *vs.* 32.1%; *p* = 0.768) was not statistically different between the two groups. There was no difference in BMI between the two groups (23.61 ± 3.23 kg/m^2^ *vs.* 24.35 ± 4.16 kg/m^2^; *p* = 0.462). Similarly, fasting blood glucose (*p* = 0.520), serum total cholesterol (*p* = 0.629), and triglyceride levels (*p* = 0.450) were not different between the two groups. Because patients in the HCV-uninfected group were significantly older (*p* = 0.003), we cannot exclude the possibility that age might be a confounding factor when assessing the prevalence of diabetes and hypertension.

**Figure 1:**
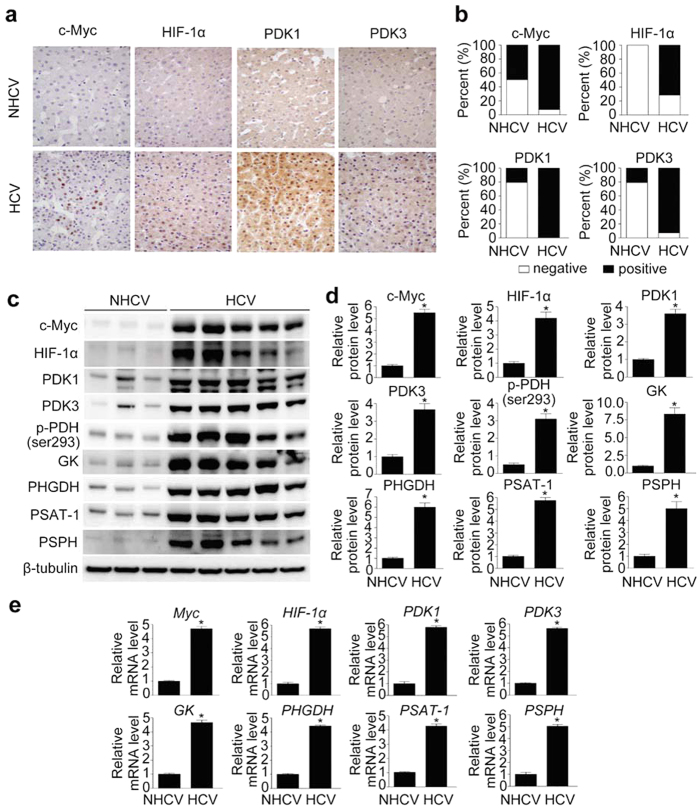
HCV infection increases c-Myc, HIF-1α, PDK1, and PDK3 levels in human liver. (**a**) Representative images of immunohistochemical (IHC) analysis of c-Myc, HIF-1α, PDK1, and PDK3 levels in HCV-non-infected (NHCV) and HCV-infected (HCV) human liver sections. Images of one representative NHCV sample and one HCV liver sample are shown. (**b**) Comparison of c-Myc, HIF-1α, PDK1, and PDK3 expression in liver sections from NHCV (n = 14) and HCV (n = 14) subjects. IHC signals corresponding to c-Myc, HIF-1α, PDK1, and PDK3 were scored as negative (<5%) or positive (>5%) based on the estimated fraction of stained hepatic tissue showing moderate-to-high signal intensity. (**c**) Representative immunoblot of c-Myc, HIF-1α, PDK1, PDK3, phospho-PDH Ser293, GK, PHGDH, PSAT-1, and PSPH in NHCV and HCV human liver tissue. β-tubulin was analyzed as an internal control. (**d**) Relative protein abundances of c-Myc, HIF-1α, PDK1, PDK3, phospho-PDH Ser293, GK, PHGDH, PSAT-1, and PSPH in NHCV and HCV human liver tissue (n = 7 in each group). Immunoblot band intensities are expressed as means ± SEM of three independent experiments. β-tubulin was analyzed as an internal control. *P < 0.001 compared with NHCV. (**e**) Representative SYBR real-time RT-PCR analysis of expression levels of Myc, HIF-1α, PDK1, PDK3, GK, PHGDH, PSAT-1, and PSPH in normal and HCV-infected human liver tissue. GAPDH mRNA was used as an internal control. Data are means ± SEM of three independent measurements (n = 7 in each group). *P < 0.001 compared with NHCV.

IHC revealed that the majority of HCV-infected liver tissues were positive for c-Myc (13/14) and hypoxia-inducible factor-1α (HIF-1α) (10/14), and nearly all were positive for PDK1 and PDK3, whereas only a few (3/14) of HCV-uninfected livers were IHC-positive for PDK1 and PDK3 (*p* < 0.001), and none were HIF-1α positive (*p* < 0.001; Fig. 1a,b). In addition, the relative proportion of c-Myc-positive patients (7/14) was significantly smaller in HCV-uninfected controls (*p* = 0.021; Fig. 1a,b). To examine whether expression of these enzymes differed according to viral genotype, HCV-infected liver tissues were grouped according to genotype (data obtained from medical records). The majority were infected with HCV genotype 1b (n = 8) or genotype 2a (n = 4), while one was infected with genotype 3a; one tissue remained unclassified. Regardless of genotype, these tissues tended to show a robust increase in expression of the indicated enzymes when compared with HCV-uninfected tissues (Table 2). Nevertheless, direct comparison among genotypes could not be done because the number of samples infected with genotype 3a was too small.

To further analyze metabolic reprograming in HCV-infected liver, we compared protein and mRNA expression levels of glycolytic enzymes between a representative set of 14 biopsied liver tissues from HCV-positive patients and 14 from HCV-negative patients. Both protein and mRNA levels of the key glycolytic enzyme glucokinase (GK) were robustly elevated in HCV-infected human liver (Fig. 1c–e). Because c-Myc and HIF-1α play a predominant role in inducing glycolysis^29^, we also examined their expression. Levels of both c-Myc and HIF-1α were markedly increased in HCV-infected liver (Fig. 1c–e). In cancer cells, c-Myc and HIF-1α act as a driving force that facilitates aerobic glycolysis^29^, mediated in part by induction of PDK1^29^,^30^. To determine whether this metabolic adaptation also occurs in HCV-infected liver tissue, we measured protein and mRNA expression levels of PDK isoenzymes, which act as gatekeepers by diverting pyruvate flux away from oxidative phosphorylation in the citric acid cycle. Expression of both PDK1 and PDK3 was also elevated in HCV-infected liver (Fig. 1c–e). In accordance with the upregulation of PDK isoenzymes, the p-Ser293 PDH protein level was significantly higher in HCV-infected liver than in uninfected liver (Fig. 1c,d). These findings imply that the elevated levels of glycolytic intermediates resulting from upregulation of glycolysis in HCV-infected hepatocytes are utilized for purposes other than mitochondrial respiration. Considering the role of serine and glycine biosynthesis in cancer cells, we hypothesized that enhanced glycolysis may contribute to increased serine biosynthesis pathway in HCV-infected liver. Consistent with this idea, protein and mRNA expression levels of PHGDH, PSAT-1, and PSPH were also elevated (Fig. 1c–e).

### Infection with HCV upregulates enzymes involved in glycolysis and serine biosynthesis in Huh-7.5 cells

**Figure 2:**
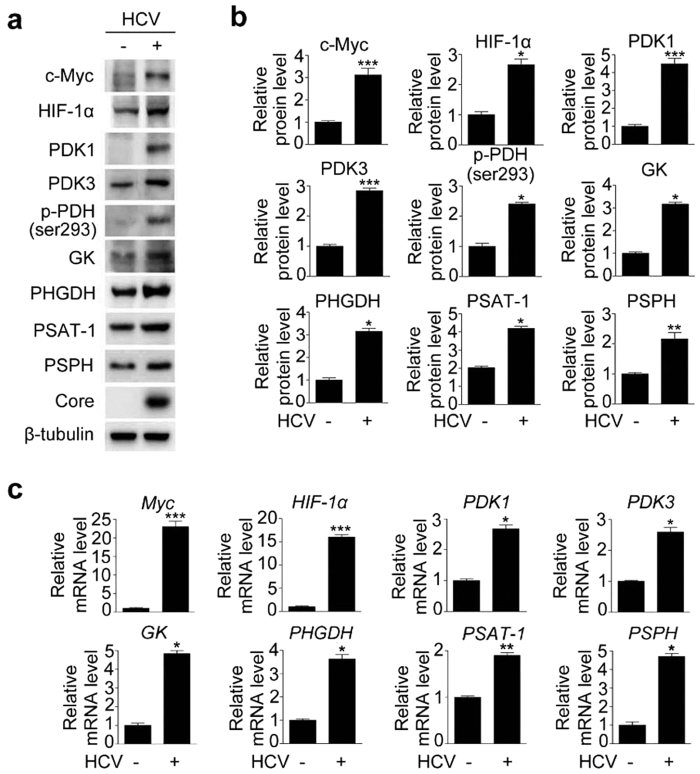
HCV infection increases the levels of enzymes involved in glycolysis and serine biosynthesis in human liver and Huh-7.5 cells. (**a**) Representative immunoblot of c-Myc, HIF-1α, PDK1, PDK3, phospho-PDH Ser293, GK, PHGDH, PSAT-1, and PSPH levels in JFH-1-infected cells. β-tubulin was analyzed as an internal control. (**b**) Relative levels of c-Myc, HIF-1α, PDK1, PDK3, phospho-PDH Ser293, GK, PHGDH, PSAT-1, and PSPH in JFH-1-infected cells. Immunoblot band intensities are expressed as means ± SEM of three independent experiments. β-tubulin was analyzed as an internal control. *P < 0.01, **P < 0.05, ***P < 0.001 compared with uninfected control cells. (**c**) Representative SYBR real-time RT-PCR analysis of expression levels of c-Myc, HIF-1α, PDK1, PDK3, GK, PHGDH, PSAT-1, and PSPH in JFH-1-infected cells. GAPDH mRNA was used as an internal control. Data are means ± SEM of three independent measurements from three separate experiments. *P < 0.01, **P < 0.05, ***P < 0.001 compared with uninfected control cells.

To determine the direct effects of HCV on the expression of glycolytic enzymes, we investigated whether mRNA and protein levels of enzymes involved in glycolysis and serine biosynthesis are altered in HCC Huh-7.5 cells infected with hepatitis C virus genotype 2a (JFH-1). In accordance with findings in human liver, JFH-1-infected Huh-7.5 cells contained higher mRNA and protein levels of c-Myc and HIF-1α (Fig. 2a–c). In addition, these cells expressed higher levels of PDK1 and PDK3 mRNA and protein (Fig. 2a–c). These net changes in PDK isoenzymes in turn increased the level of p-Ser293 PDH, which favors facilitated glycolysis (Fig. 2a,b). Moreover, consistent with the human data, protein and mRNA levels of PHGDH, PSAT-1, and PSPH were elevated (Fig. 2a–c). Collectively, these data strongly indicate that, in HCV-infected hepatocytes, elevated glycolysis, together with elevated p-Ser293 PDH, permits upregulation of serine biosynthetic pathways.

### Pharmacological inhibition of c-Myc and PDK blocks HCV replication in HCV-infected Huh 7.5 cells

**Figure 3:**
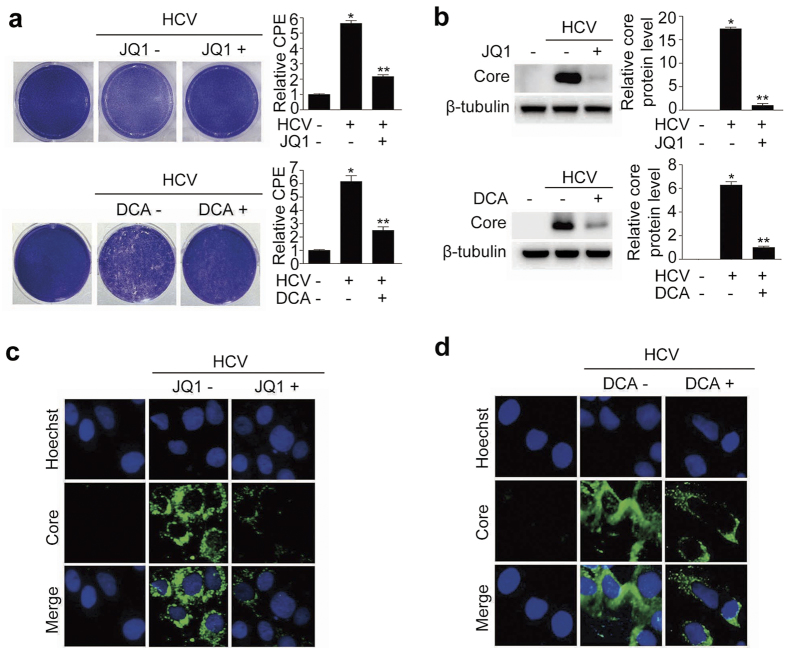
Pharmacological inhibition of c-Myc and PDK blocks HCV replication. (**a**) Representative images of plaque assay of JFH-1-infected cells treated with or without JQ1 (500 nM) or DCA (10 mM) for 3 days. Occurrence of cytopathic effect (CPE) was examined using extraction solution (methanol) and measuring absorbance at 540 nm. Data were expressed as fold increases in CPE relative to the uninfected control cells (bar graph). (**b**) Representative immunoblot of HCV core protein abundance in JFH-1-infected cells with or without JQ1 or DCA treatment. Immunoblot band intensities are expressed as means ± SEM of three independent experiments. β-tubulin levels were analyzed as an internal control. (**c,d**) Representative images of immunofluorescence detection of HCV core protein in JFH-1-infected cells treated with or without JQ1 (**c**) or DCA (**d**). Cells were immunostained with anti-HCV core antibody (green), and nuclei were stained with Hoechst (blue).

In light of the elevated expression of c-Myc and PDKs in HCV-infected hepatocytes and human liver tissue, we conducted plaque assays to determine whether pharmacologic inhibition of these proteins could determine the fate of HCV replication. JQ1 is a selective small-molecule bromodomain inhibitor^31^ that has therapeutic effects at a dose of 0.2–1 μM in several *in vitro* cancer models^32^,^33^,^34^. DCA is a pan-inhibitor of several PDK isoforms and exerts its inhibitory effects by binding to an allosteric site within the N-terminal domain^35^. A larger dose of DCA is generally required, for example, 5–10 mM, to achieve therapeutic efficacy in cancers with high lactate concentrations^36^,^37^. The median effective concentration (EC_50_) of DCA required for the inhibition of HCV replication was 4.394 mM (Supplementary Fig. S1). Therefore, we treated cells with 500 nM JQ1 and 10 mM DCA, based on our finding that increased glycolysis is a major metabolic characteristic in HCV-infected hepatocytes. The number of plaques (appearing as white dots) was markedly increased by HCV infection, but dramatically diminished by treatment with the c-Myc inhibitor JQ1 or the PDK inhibitor DCA, as demonstrated by a reduction in the number of plaques (Fig. 3a). In addition, the amount of core protein was decreased by these pharmacological inhibitors, as revealed by immunoblotting and immunofluorescence (Fig. 3b–d). We also found that HCV copy number was significantly decreased by these compounds, as determined by quantitative RT-PCR (Supplementary Fig. S2a,b). To confirm that inhibition of HCV replication was not attributable to chemical-induced cytotoxicity, we next performed a cell viability assay using Cell Counting Kit-8 (CCK-8). Both JQ1 (500 nM) and DCA (10 mM) inhibited HCV replication but did not affect cell viability (Supplementary Fig. S2c,d). Taken together, these findings indicate that activation of each of these signaling pathways in host cells is required for proliferation of HCV, and that pharmacological inhibition of glycolysis perturbs viral replication.

### Inhibition of c-Myc and PDK represses a cascade of enzymes responsible for glycolysis and serine biosynthesis in HCV-infected hepatocytes

**Figure 4:**
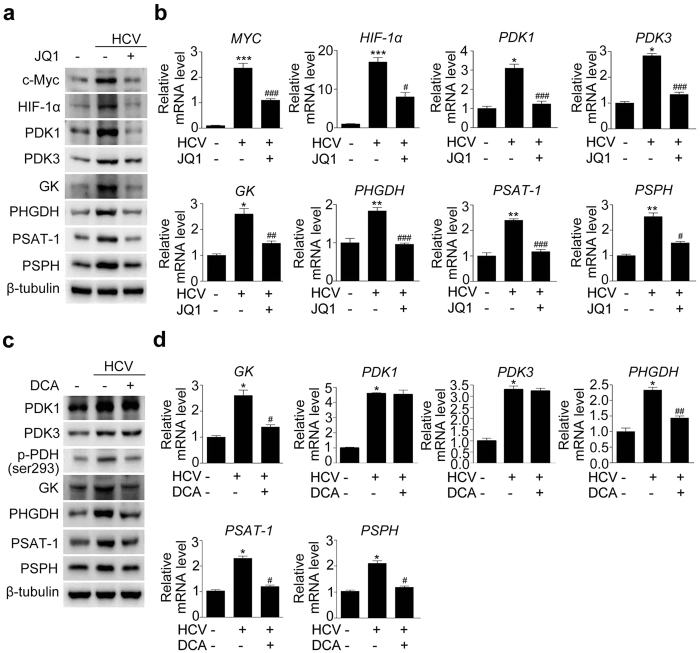
Pharmacological inhibition of c-Myc and PDK reduces the mRNA and protein levels of enzymes involved in glycolysis and serine synthesis in JFH-1-infected Huh7.5 cells. (**a**) Representative immunoblot showing c-Myc, HIF-1α, PDK1, PDK3, GK, PHGDH, PSAT-1, and PSPH levels in JFH-1-infected cells treated with JQ1 (500 nM for 3 days). (**b**) Representative SYBR real-time RT-PCR analysis of the mRNA expression levels of c-Myc, HIF-1α, PDK1, PDK3, GK, PHGDH, PSAT-1, and PSPH in JFH-1-infected cells treated with or without JQ1 (500 nM, 3 days). GAPDH mRNA was used as an internal control. Data are means ± SEM of three independent measurements from three separate experiments. *P < 0.01, **P < 0.05, ***P < 0.001 compared with uninfected control cells; ^#^P < 0.01, ^##^P < 0.05, ^###^P < 0.001 compared with HCV infection alone. (**c**) Representative immunoblot showing PDK1, PDK3, phospho-PDH Ser293, GK, PHGDH, PSAT-1, and PSPH levels in JFH-1-infected cells treated with DCA (10 mM for 3 days). (**d**) Representative SYBR real-time RT-PCR analysis of the mRNA expression levels of GK, PHGDH, PSAT-1, and PSPH in JFH-1-infected cells treated with or without DCA. GAPDH mRNA was used as an internal control. Data are the means ± SEM of three independent measurements from three separate experiments. *P < 0.01, **P < 0.05, ***P < 0.001 compared with uninfected control cells; ^#^P < 0.01, ^##^P < 0.05, ^###^P < 0.001 compared with HCV infection alone.

To obtain a better understanding of the molecular mechanism by which c-Myc and PDK modulates HCV replication, we examined expression of enzymes involved in glycolysis and serine biosynthesis. Protein and mRNA level of c-Myc, which was markedly increased in HCV-infected hepatocytes, was attenuated following JQ1 treatment (Fig. 4a,b and Supplementary Fig. S3a). The elevated protein levels of GK, PHGDH, PSAT-1, and PSPH observed in HCV-infected hepatocytes reverted to almost normal levels upon JQ1 treatment (Fig. 4a and Supplementary Fig. S3a). This finding correlated well with the mRNA expression of these enzymes (Fig. 4b). Of note, pharmacological inhibition of c-Myc repressed HIF-1α, PDK1, and PDK3 protein and mRNA levels, suggesting that c-Myc inhibition can alter overall cellular metabolism to disfavor glycolysis in HCV-infected hepatocytes.

Next, we repeated the same experiment using DCA instead of JQ1. Although DCA inhibits the activity of all four PDK isoforms, it does not regulate transcription or translation of the enzymes. Therefore, it is generally accepted that reduced the amount of phospho-PDHE1α protein reflects the ability of a compound to inhibit PDK. Consistent with this notion, our data showed that PDK1/3 mRNA or protein levels were not decreased by DCA, despite a significant decrease of Ser293phospho-PDH level (Fig. 4c,d and Supplementary Fig. S3b). As in JQ1-treated hepatocytes, mRNA and protein levels of PHGDH, PSAT-1, and PSPH, as well as GK, were significantly reduced (Fig. 4c,d and Supplementary Fig. S3b).

**Figure 5:**
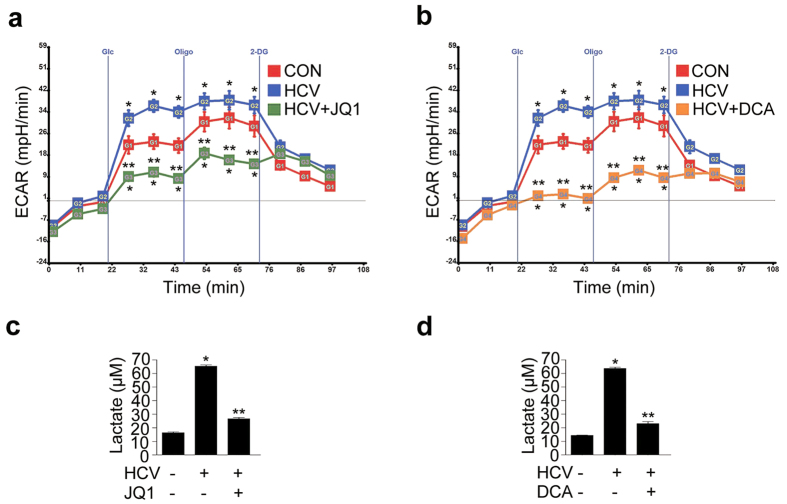
Pharmacological inhibition of c-Myc and PDK reduces glycolysis-derived lactate production in JFH-1 infected Huh7.5 cells. (**a**) Extracellular acidification rates (ECAR) in JFH-1-infected Huh 7.5 cells treated with JQ1 (500 nM) for 72 hr. *P < 0.001 compared with uninfected control cells; **P < 0.001 compared with HCV infection alone. Glc, Glucose; Oligo, oligomycin; 2DG, 2-deoxyglucose. (**b**) ECAR in JFH-1-infected Huh 7.5 cells treated with DCA (10 mM) for 72 hr. *P < 0.001 compared with uninfected control cells; **P < 0.001 compared with HCV infection alone. (**c**) Lactate levels in JFH-1-infected Huh 7.5 cells treated with JQ1 (500 nM) for 72 hr. *P < 0.001 compared with uninfected control cells; **P < 0.001 compared with HCV infection alone. (**d**) Lactate levels in JFH-1-infected Huh7.5 cells treated with DCA (10 mM) for 72 hr. *P < 0.001 compared with uninfected control cells; **P < 0.001 compared with HCV infection alone.

To further confirm that the increased glycolytic flux in HCV-infected hepatocytes was actually attenuated by these inhibitors, we measured the extracellular acidification rate (ECAR) and lactate level in JFH-1-infected or -uninfected Huh 7.5 cells. The ECAR significantly increased upon HCV infection and was significantly reduced by JQ1 and DCA (to levels lower than those observed in JFH-1-uninfected Huh 7.5 cells) (Fig. 5a,b). In the same context, cellular lactate levels were also significantly lower after treatment with these compounds (Fig. 5c,d).

To exclude the possibility of off-target inhibition by JQ1, we used another c-Myc inhibitor, 10058-F4, and a siRNA targeting c-Myc. 10058-F4 (50 μM) markedly suppressed the amount of core protein, as well as c-Myc, PDK1, and PDK3 protein levels (Supplementary Fig. S4a). Again, the indicated dose was not cytotoxic, as evidenced by the CCK-8 assay (Supplementary Fig. S4a). Furthermore, knockdown of c-Myc by transfection of siRNA produced a similar result, again without inducing cellular toxicity (Supplementary Fig. S4b). This was also the case for siRNA-based double-knockdown of PDK1 and PDK3 (Supplementary Fig. S3c). From these findings, we can assume that 1) the antiviral effects of JQ1 and DCA are mediated by their molecular targets, c-Myc and PDKs, respectively; 2) that the efficacy of these compounds is likely the result of a class effect rather than an off-target effect; and 3) that the result of the *in vitro* efficacy experiment is reliable because the indicated doses of the compounds did not cause cellular toxicity.

To summarize, these observations suggest that the forced increase in PDH activity upon suppression of PDK in HCV-infected hepatocytes is sufficient to drive glucose metabolism away from aerobic glycolysis and the serine biosynthesis pathway. In addition, these data highlight a role of c-Myc as an upstream regulator of HIF-1α and PDKs, key glycolytic enzymes in HCV-infected cells.

### DCA markedly potentiates IFN-α and ribavirin-induced inhibition of HCV replication

**Figure 6:**
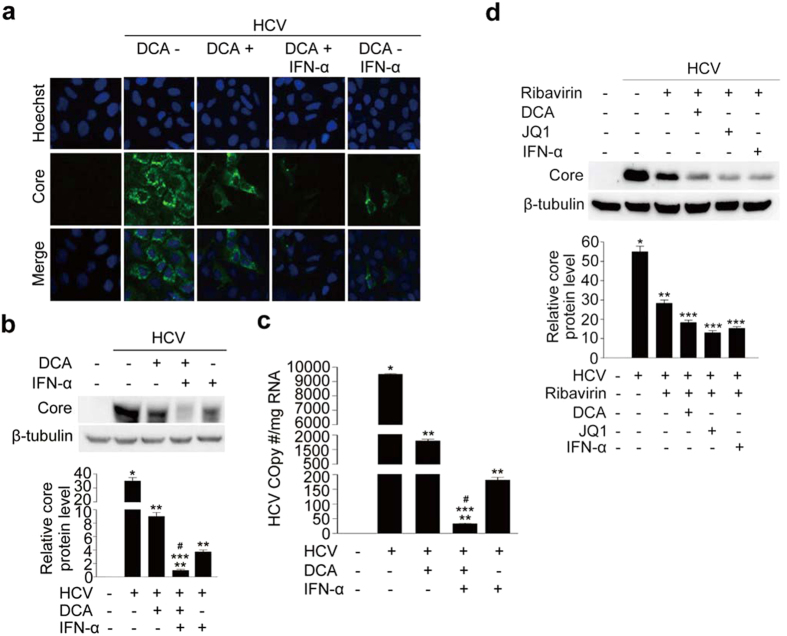
DCA potentiates the inhibitory effect of interferon (IFN)-α or ribavirin on HCV replication. (**a**) Representative images of immunofluorescence detection of HCV core protein in JFH-1-infected cells co-treated with DCA (10 mM) and IFN-α (100 IU/ml) for 72 hr. Cells were immunostained with anti-HCV core antibody (green), and nuclei were stained with Hoechst (blue). (**b**) Representative immunoblot of HCV core protein abundance in JFH-1-infected cells co-treated with DCA and IFN-α. Quantitation of immunoblot band intensities are expressed as means ± SEM of three independent experiments. β-tubulin was analyzed as an internal control. (**c**) Representative TaqMan real-time RT-PCR analysis of expression levels of the HCV core protein in JFH-1-infected cells co-treated with DCA and IFN-α. Data are means ± SEM of three independent measurements from three separate experiments. *P < 0.001 compared with uninfected control cells; **P < 0.001 compared with HCV infection alone; ***P < 0.001 compared with HCV-infected cells treated with DCA; ^*#*^*P* < 0.01 compared with HCV-infected cells treated with IFN-α. (**d**) Representative immunoblot showing HCV core protein levels in JFH-1-infected cells co-treated with DCA (10 mM), JQ1 (500 nM), or IFN-α (100 IU/ml) in the presence of ribavirin (25 μg/ml) for 3 days. Band intensities are expressed as the mean ± SEM of three independent experiments. β-tubulin was used as an internal control. *P < 0.01 compared with uninfected control cells; **P < 0.001 compared with HCV infection alone; ***P < 0.001 compared with treatment with ribavirin alone.

Finally, we investigated the inhibitory effect of DCA in combination with IFN-α, a standard component of regimens used to treat chronic hepatitis C infection, on HCV replication. DCA induced profound inhibition of HCV replication, as determined by immunofluorescence staining, consistent with the findings shown in Fig. 3 (Fig. 6a). A dose of 100 IU of IFN-α also exerted a significant antiviral effect, but when IFN-α was co-administered with DCA, the amount of core protein was further reduced (Fig. 6a). Consistent with this, core protein levels were effectively controlled by both DCA and IFN-α, as determined by immunoblotting; moreover, co-treatment with these agents further suppressed core protein levels (Fig. 6b). Quantitative RT-PCR analysis confirmed that HCV replication, determined based on viral copy number, was markedly inhibited by DCA, and further decreased upon addition of IFN-α (Fig. 6c). Furthermore, both DCA or JQ1, when used in combination with ribavirin, induced further and significant reductions in core protein levels when compared with ribavirin alone (Fig. 6d). The inhibitory effect of a combination of DCA or JQ1 and ribavirin on HCV replication was almost identical to that of a combination of IFN-α and ribavirin (Fig. 6d).

Collectively, these findings indicate that PDK inhibitors could be ideal drug candidates for counteracting HCV infection because these compounds not only negatively regulate HCV replication on their own, but also increase the inhibitory effect of IFN-α or ribavirin.

## Discussion

In this study, we found that protein and mRNA expression levels of PDKs was elevated in HCV-infected hepatocytes. Importantly, this elevated PDK activity resulted in the production of abundant glycolytic intermediates, which fuel serine and glycine synthesis. Because *de novo* synthesized serine and glycine are utilized in nucleotide synthesis, we hypothesized that these metabolic changes create a cellular milieu that favors rapid HCV replication. The PDK inhibitor DCA suppressed HCV replication in HCV-infected hepatocytes, and this suppression was accompanied by metabolic reprogramming that disfavored glycolysis and serine synthesis. In addition, c-Myc, in conjunction with HIF-1α, was upregulated in HCV-infected hepatocytes, decreasing glucose oxidation by increasing PDK levels, whereas blocking Myc with a pharmacologic inhibitor successfully reversed the HCV-induced changes in the glucose oxidation and serine biosynthetic pathways.

Recent advances in the field of virology have inspired the speculation that, in order to fulfill their demand for rapid replication, viruses delicately orchestrate host cell metabolism^18^, just as tumors cells do. In this regard, it seems that upregulation of host cell glycolysis is a universal and indispensable process in viral replication, as revealed by previous studies performed in a variety of viral strains including HCV, HCMV, HSV, HIV, Dengue virus, and adenovirus^19^,^20^,^21^,^22^,^23^,^24^,^38^,^39^. For example, a recent study showed that HCV NS5 protein directly interacts with HK, a rate-limiting enzyme of glycolysis in Huh 7.5 cells^24^. Although the mechanism has not been determined in detail, both HCMV and HSV-1 activate phosphofructokinase (PFK)-1, an enzyme mainly involved in glycolysis^23^,^40^. In this study, we found that GK and PDK responsible for glycolysis were upregulated both in HCV-infected human liver and HCV-infected hepatocytes, implying that HCV relies on host cellular energetics to successfully achieve its replication. Our study provides new evidence that HCV changes host cell metabolism to favor elevated glycolysis and serine biosynthesis. This phenomenon is driven by increased expression of PDK, which halts conversion of pyruvate to acetyl-coA in the mitochondria. Furthermore, pharmacologic inhibition of PDK was sufficient to downregulate the cascade of enzymes involved in serine synthesis. Therefore, this finding suggests that the serine biosynthetic pathway might also play important roles in HCV replication. Indeed, this finding is supported by a very recent expression array study showing that PSAT1 and PSPH levels were 1.8-fold and 1.4-fold higher, respectively, in an HCV-persistently-infected cell line than in HCV-uninfected Huh-7.5 cells^26^.

Several lines of evidence indicate that c-Myc expression is activated by HCV infection^41^,^42^. c-Myc expression increases in hepatic tissues from HCV-infected patients as well as in an HCV-transgenic mouse model, and Akt activation by NS5A following stabilization of β-catenin is responsible for inducing c-Myc transcription^41^. Given the importance of c-Myc in carcinogenesis, c-Myc may play a critical role in HCV-associated HCC^41^. Consistent with these findings, we observed an increase in c-Myc expression in HCV-infected tissues and in HCV-infected hepatocytes; we also observed that increased c-Myc expression is implicated in viral replication via host cell metabolic reprogramming, as evidenced by the siRNA and inhibitor experiments. A more recent study showed that the gene product of adenovirus E4ORF1 binds to Myc in the host cell nucleus, increasing transcription of glycolytic enzymes such as HK2 and PF-1, and promoting viral replication^39^. Similarly, HIF-1α also plays an important role in virus-induced host cell adaptation. Metabolic adaptations to HCV may contribute to stabilization of HIF-1α in HCV-infected cells even under normoxic conditions^43^. This stabilized HIF-1α further induces glycolytic enzymes, thus forming a positive feedback loop^43^. Consistent with these findings, the results described here show that levels of c-Myc, HIF-1α, and PDKs were simultaneously elevated in HCV-infected human liver and Huh-7.5 cells. Besides, c-Myc inhibitor suppressed protein levels of both p-Ser293 PDH and HIF-1α, suggesting that the actions of these enzymes are intertwined and that they work collaboratively in a complex manner to promote glycolysis. The precise mechanism by which each of these factors regulates the others remains to be determined.

Our results revealed that DCA effectively blocked HCV replication in HCV-infected Huh-7.5 cells. Furthermore, it further inhibited HCV replication when co-administered with IFN-α. The dose-related adverse effects of IFN-α are the primary causes of unintended discontinuation of the drug^44^. Consequently, over the last decade, traditional IFN-α has been largely replaced by its PEGylated form, which has a longer half-life and therefore enables once-a-week treatment; however, despite the improved safety profile, concerns about adverse effects persist in clinical practice. Our data demonstrate that clinical application of PDK inhibitors against chronic hepatitis C could reduce the required dosage of PEGylated IFN-α, thereby ameliorating or abolishing adverse effects. Although DCA is effective in humans with severe lactic acidosis and glioblastoma multiforme (GBM)^10^,^45^, its clinical application is accompanied by adverse effects. These include mild-to-severe peripheral neuropathy, which is reversible and resolves gradually after discontinuation of the drug^46^. The most critical adverse effect of DCA is liver cancer, which makes human application impossible^47^. Thus, efficacious and safer novel PDK inhibitors are being developed to overcome these adverse effects^48^,^49^,^50^,^51^; to date, however, none of them have reached phase III clinical trials, and in any case these drugs are mainly being investigated in the context of either diabetes or cancer^48^,^49^,^50^,^51^,^52^,^53^. Our study demonstrates that these drugs could widen their indications to include chronic hepatitis C. Although JQ1 was effective in preclinical animal models, its clinical application is limited due to its short (1 hr) half-life. In addition, the selectivity of the compound means that it may have off-target effects; for example, JQ1 inhibits BRD2/3/4^54^. The ideal c-Myc inhibitor would be specific for BRD4, since BRD4 mainly regulates the transcription of c-Myc^54^. A very recent study involving JQ1 revealed that BRD4 is a novel therapeutic target in cases of liver fibrosis^55^. Taken together with our findings, these data suggest that a c-Myc inhibitor that specifically targets BRD4 would be a novel candidate treatment for HCV and its associated complications.

Although recently developed direct-acting antivirals have shown improved sustained virologic responses and are generally well tolerated with less side effects than those seen with PEGylated IFN-α and ribavirin, drug resistance still continues to be a problem. For example, baseline mutation at the Y93 site of NS5A protein was detected in 13 of 147 HCV genotype 3-infected patients in a clinical trial of daclatasvir plus sofosbuvir; the mutation was associated with daclatasvir resistance and lower sustained virologic response rates^56^. The mutation also emerged in 9 of 16 patients with relapse^56^. Likewise, the Q80K mutation in NS3/4A protease is associated with reduced susceptibility to simeprevir^57^. In addition, up to one fourth of patients taking these novel direct-acting antivirals experience headache and fatigue^56^,^58^. The present study suggested that targeting the host-virus metabolic interaction involved in HCV replication using small molecule inhibitors of PDK has the potential to overcome resistance to direct-acting antivirals. Therefore, it will be necessary to investigate whether combinations of PDK inhibitors with these antivirals can reduce the dosage or duration of treatments with these drugs and thereby reduce the potential adverse effects of these antivirals in clinical practice.

In summary, our study demonstrated a new role for PDKs in the regulation of HCV, which reprograms host cell metabolism to favor increased glycolysis and *de novo* serine biosynthesis, which in turn increases synthesis of the nucleotides that fuel HCV replication. By decreasing PDK activity, DCA treatment of HCV-infected cells reversed the activities of enzymes involved in both serine synthesis and glycolysis. Notably, altering the cellular environment to disfavor aerobic glycolysis, without directly targeting HCV itself, exerted a significant inhibitory effect on replicative activity. This study broadens our understanding of pathophysiology, expands the scope of treatment strategies of HCV, and raises the possibility of identifying additional novel targets.

## Materials and Methods

### Patients and specimens

Immunohistochemical (IHC) studies were performed to compare c-Myc, HIF-1α, and PDK expression between HCV-infected and HCV-uninfected human liver tissue. HCV-infected human liver tissues were obtained from 14 patients serologically diagnosed as HCV-infected, as determined by the presence of HCV antibody, at Keimyung University Dongsan Hospital in Daegu, Korea, between 2006 and 2012. HCV-uninfected liver tissues were collected from 14 patients with no serologic evidence of HCV antibodies: nine with cholestatic hepatitis, three with regenerative liver nodules, and two with steatohepatitis.

To evaluate metabolic reprogramming in HCV-infected liver, mRNA and protein expression of enzymes involved in glycolysis and serine biosynthesis were measured in 14 samples of HCV-positive liver tissue obtained by needle biopsy and compared with those in 14 HCV-negative liver tissue taken from six HCC patients, six cholangiocarcinoma patients, one case of breast cancer with hepatic metastasis, and one patient with choledocholithasis, all of whom underwent surgical hepatic resection at Kyungpook National University Hospital in Daegu, Korea, between 2011 and 2013. The study protocol was approved by the clinical research ethics committee of the hospital.

### Immunohistochemical (IHC) staining and scoring

Human liver sections (4 μm) were used for IHC staining. IHC staining was performed using an automated system (Autostainer 360, Lab Vision, Fremont, CA, USA). The primary antibodies used in this investigation were against c-Myc (1:200, Abcam, Cambridge, UK), HIF-1α (1:150, Novus Biologicals, Littleton, CO, USA), PDK1 (1:100, Enzo, East Farmingdale, NY, USA), and PDK3 (1:200, Novus Biologicals). Expression was scored as positive if >5% of hepatocytes showed moderate-to-high signal intensity.

#### Cell culture and reagents

See Supplementary Materials and Methods.

### HCV stock preparation

The JFH-1 strain (genotype 2a) of HCV was produced as described previously^59^.

### HCV infection

Huh-7.5 cells were infected at a multiplicity of infection (MOI) of 0.01, and then subcultured every 3–4 days. When over 80% of cells were infected, cells were treated with DCA (10 mM, 3 days) or JQ1 (500 nM, 3 days). To determine whether co-treatment with INF would further inhibit DCA-induced HCV replication, 100 IU/ml recombinant IFN-α 2b was co-administered with DCA for 3 days.

#### Immunofluorescence analysis

See Supplementary Materials and Methods.

#### Immunoblotting analysis

See Supplementary Materials and Methods.

#### Determination of the EC_50_ of DCA required for the inhibition of HCV replication

See Supplementary Materials and Methods.

### Plaque assay

Huh-7.5 cells were infected at an MOI of 0.01. Infected cells were overlaid with culture medium containing 0.8% methylcellulose, and then treated with DCA or JQ1. Cells were fixed with buffered formalin and stained with crystal violet (Sigma). Formation of cytopathic plaques was visualized by staining the cell monolayers with 0.08% crystal violet solution. One-third to one-half of total well volume of 100% methanol was added to each sample. One hundred microliters of each sample was transferred to a 96-well microtiter plate, and the absorbance was measured at an OD of 540 nm in a plate reader.

### Measurement of the extracellular acidification rate and performance of the lactate assay

JFH-1-infected Huh7.5 cells (5 × 104) were seeded in an XF assay plate and treated with DCA (10 mM) for 3 days or with JQ1 (500 nM) for 3 days. The medium was then exchanged for assay medium (XF base medium) and the cells were incubated in a non-CO2 incubator at 37 °C for 1 h before the assay. Glucose (10 mM), oligomycin (2 μM final concentration), and 2-DG (50 mM final concentration) were diluted in assay medium and injected into ports A, B, and C, respectively, of a Seahorse analyzer (Seahorse Bioscience, Billerica, MA, USA). The machine was calibrated and the assay was performed using the glycolytic stress test assay protocol, as suggested by the manufacturer. Addition of glucose defines glycolysis, oligomycin represents maximum glycolytic capacity, and 2-DG represents acidification associated with non-glycolytic activity. To measure lactate levels, JFH-1-infected Huh7.5 cells were treated with DCA or JQ1. Samples from three independent experiments were assayed using a lactate assay kit (BioVision, Mountain View, CA), according to the manufacturer’s instructions.

### Measurement of cell proliferation and cytotoxicity

Cytotoxicity was measured using Cell Counting Kit-8 (CCK-8) (Dojindo Lab., Kumamoto, Japan). Briefly, cells (5 × 104 cells/well) were seeded in a 96-well plate and cultured. Next, 10 μl of CCK-8 solution was added to each well and the plate was incubated at 37 °C for 3 h. The optical density (OD) at 450 nm was then measured in Versa Max microplate reader (Molecular Devices, CA).

### Small interfering RNAs (siRNAs) targeting c-Myc, PDK1, and PDK3

Pre-designed siRNAs targeting c-Myc, PDK1, and PDK3 were purchased from Bioneer (Daejeon, Korea). Cells were transfected for 5 h with 100 nM of siRNA using the Lipofectamine RNAiMAX reagent (Invitrogen, Carlsbad, CA, USA), cultured in medium containing 0.5% FBS, and harvested approximately 72 h post-transfection. The effects of siRNAs against c-Myc, PDK1, and PDK3 on the expression of endogenous c-Myc, PDK1, and PDK3 were assessed by immunoblotting.

#### Quantitative and SYBR Real-time RT-PCR

See Supplementary Materials and Methods.

### Ethics statement

This study was approved by the Institutional Review Board of Kyungpook National University Hospital (IRB no. KNUMCBIO_12-1007) and Keimyung University Dongsan Hospital (IRB no. 11-256 and IRB no. 13-16). The study protocol was approved by the clinical research ethics committee of the hospital and all experiments were performed in accordance with approved guidelines. Informed written consent was obtained from each patient.

### Statistical analyses

Clinical data management and statistical analyses were performed using SPSS ver. 18.0 (SPSS, Chicago, IL). Data are presented as means ± SEM. Associations between protein expressions were evaluated using Pearson’s χ^2^ test. The prevalence of comorbidities such as diabetes and hypertension in chronic hepatitis C patients and control patients was also compared using Pearson’s χ^2^ test. Numerical parameters such as age, height weight, body mass index (BMI), and serum parameters were evaluated using Student’s t-test. *p* < 0.05 was considered significant.

## Additional Information

**How to cite this article**: Jung, G.-S. *et al*. Pyruvate dehydrogenase kinase regulates hepatitis C virus replication. *Sci. Rep.* **6**, 30846; doi: 10.1038/srep30846 (2016).

## Supplementary Material

Supplementary Information

## Figures and Tables

**Table 1:** Metabolic features of patients with or without HCV infection.

	HCV (N = 28)	NHCV (N = 28)	*P* value
Anthropometric parameter			
Age (years)	51.14 ± 11.18	61.03 ± 12.80	0.003
Height (cm)	165.08 ± 9.12	162.01 ± 10.34	0.244
Weight (kg)	64.50 ± 11.05	63.64 ± 13.28	0.984
BMI (kg/m^2^)	23.61 ± 3.23	24.35 ± 4.16	0.462
Comorbidities			
Hypertension (N, %)	7 (25.0%)	9 (32.1%)	0.768
Diabetes (N, %)	9 (32.1%)	7 (25.0%)	0.768
Biochemical parameter			
Fasting glucose (mg/dL)	111.38 ± 17.34	120.71 ± 64.05	0.520
Total cholesterol (mg/dL)	165.59 ± 54.52	158.90 ± 47.03	0.629
Triglycerides (mg/dL)	104.90 ± 55.81	127.60 ± 49.76	0.450

Data are expressed as the mean ± SD or n (%) unless otherwise indicated. HCV, HCV-infected patient; NHCV, HCV-non-infected patient; BMI, body mass index.

**Table 2:**
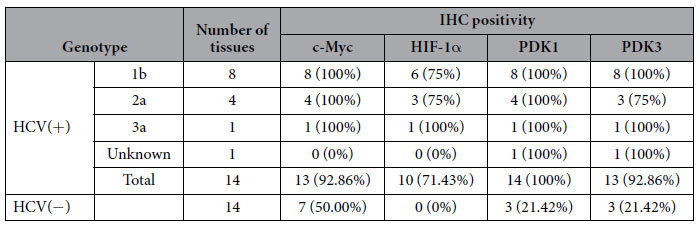
The relative prevalence of HCV genotypes and their association with the expression of key glycolytic enzymes.
