# Supplementary material for: Pyruvate dehydrogenase kinase regulates hepatitis C virus replication
Source: Sci Rep. 2016 Jul 29;6:30846. doi: 10.1038/srep30846 (PMC4965757; doi:10.1038/srep30846)
Supplement: Supplementary Information [file srep30846-s1.pdf]

Pyruvate dehydrogenase kinase regulates hepatitis C virus replication

Gwon-Soo Jung[1,2*], Jae-Han Jeon[1,2*], Yeon-Kyung Choi[1,2], Se Young Jang[1], Soo Young Park[1], Sung-Woo Kim[1,2], Jun-Kyu Byun[2], Mi-Kyung Kim[3], Sungwoo Lee[4], Eui-Cheol Shin[5], In-Kyu Lee[1,2], Yu Na Kang[6,**], and Keun-Gyu Park[1,2,**]

Gwon-Soo Jung, mine9240@naver.com; Jae-Han Jeon, ggoloo@hanmail.net; Yeon-Kyung Choi, exc4932@hanmail.net; Se Young Jang, magnolia1103@naver.com; Soo Young Park, psyoung0419@gmail.com; Sung-Woo Kim, sungwoocap@naver.com; Jun-Kyu Byun, byun0504@hotmail.com; Mi-Kyung Kim, mdkmk@dsmc.or.kr; Sungwoo Lee, swlee@dgmif.re.kr; Eui-Cheol Shin, euicheols@kaist.ac.kr; In-Kyu Lee, leei@knu.ac.kr; Yu Na Kang, yunakang@dsmc.or.kr; Keun-Gyu Park, kpark@knu.ac.kr

[1]Department of Internal Medicine, Kyungpook National University School of Medicine, Daegu, Republic of Korea

[2]Leading-edge Research Center for Diabetes and Metabolic Disease, Kyungpook National University Hospital, Daegu, Republic of Korea

[3]Department of Internal Medicine, Keimyung University School of Medicine, Daegu, Republic of Korea

[4]New Drug Development Center, Daegu-Gyeongbuk Medical Innovation Foundation, Daegu, Republic of Korea

[5]Laboratory of Immunology and Infectious Diseases, Graduate School of Medical Science and Engineering, KAIST, Daejeon, Republic of Korea

[6]Department of Pathology, Keimyung University School of Medicine, Daegu, Republic of Korea

*These authors contributed equally.

Keywords: aerobic glycolysis, dichloroacetate, hepatitis C virus, pyruvate dehydrogenase kinase

****Corresponding authors:**
Keun-Gyu Park, MD, PhD: Department of Internal Medicine, Kyungpook National University School of Medicine, 130 Dongdeok-ro, Jung-gu, Daegu, 41944, Republic of Korea. Tel.: +82-53-200-6953, Fax: +82-53-426-6722, E-mail: kpark@knu.ac.kr

Yu Na Kang, MD, PhD: Department of Pathology, Keimyung University School of Medicine, Daegu, 700-712, South Korea. Tel: +82-53-250-7036, Fax: +82-53-250-7211, E-mail: yunakang@dsmc.or.kr

Supplementary Figures

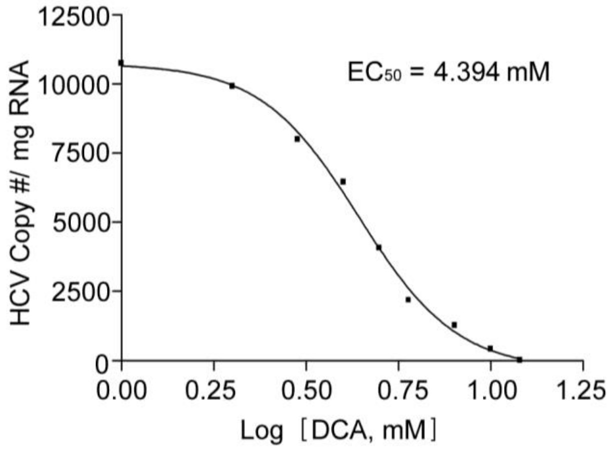

Supplementary Fig. S1. The EC50 of DCA for inhibition of HCV replication

The curve is shown as a dotted line and indicates the dose–response curve for DCA inhibition of HCV replication in JFH-1-infected cells treated with DCA (1–12 mM) for 3 days. The dose–response curve was obtained by performing non-linear regression using GraphPad Prism software after performing quantitative real-time RT-PCR.

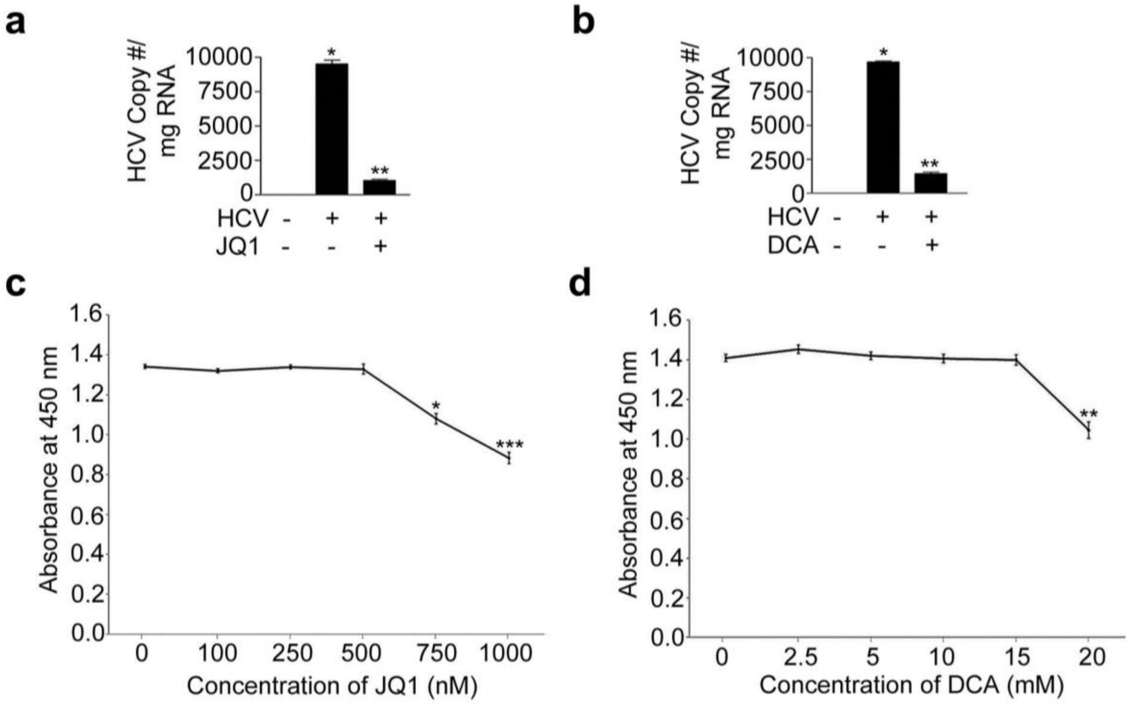

Supplementary Fig. S2. Pharmacological inhibition of c-Myc or PDK blocks HCV replication in JFH-1-infected Huh7.5 cells without inducing cytotoxicity.

(a-b) Representative TaqMan real-time RT-PCR analysis of HCV core protein expression in JFH-1-infected Huh-7.5 cells in the presence or absence of JQ1 (a) or DCA (b). Data are expressed as the mean ± SEM of three independent measurements from three separate experiments. $^{*}P < 0.001$ compared with uninfected control cells; $^{**}P < 0.001$ compared with HCV infection alone. (c-d) Cytotoxicity of JQ1 (c) and DCA (d) as measured by CCK-8 colorimetry. Huh7.5 cells were cultured in the absence of JFH-1 and treated with the indicated doses of JQ1 and DCA, respectively. Data are expressed as the mean ± SEM of three independent measurements. $^{*}P < 0.05$, $^{**}P < 0.01$, and $^{***}P < 0.001$ compared with untreated control cells.

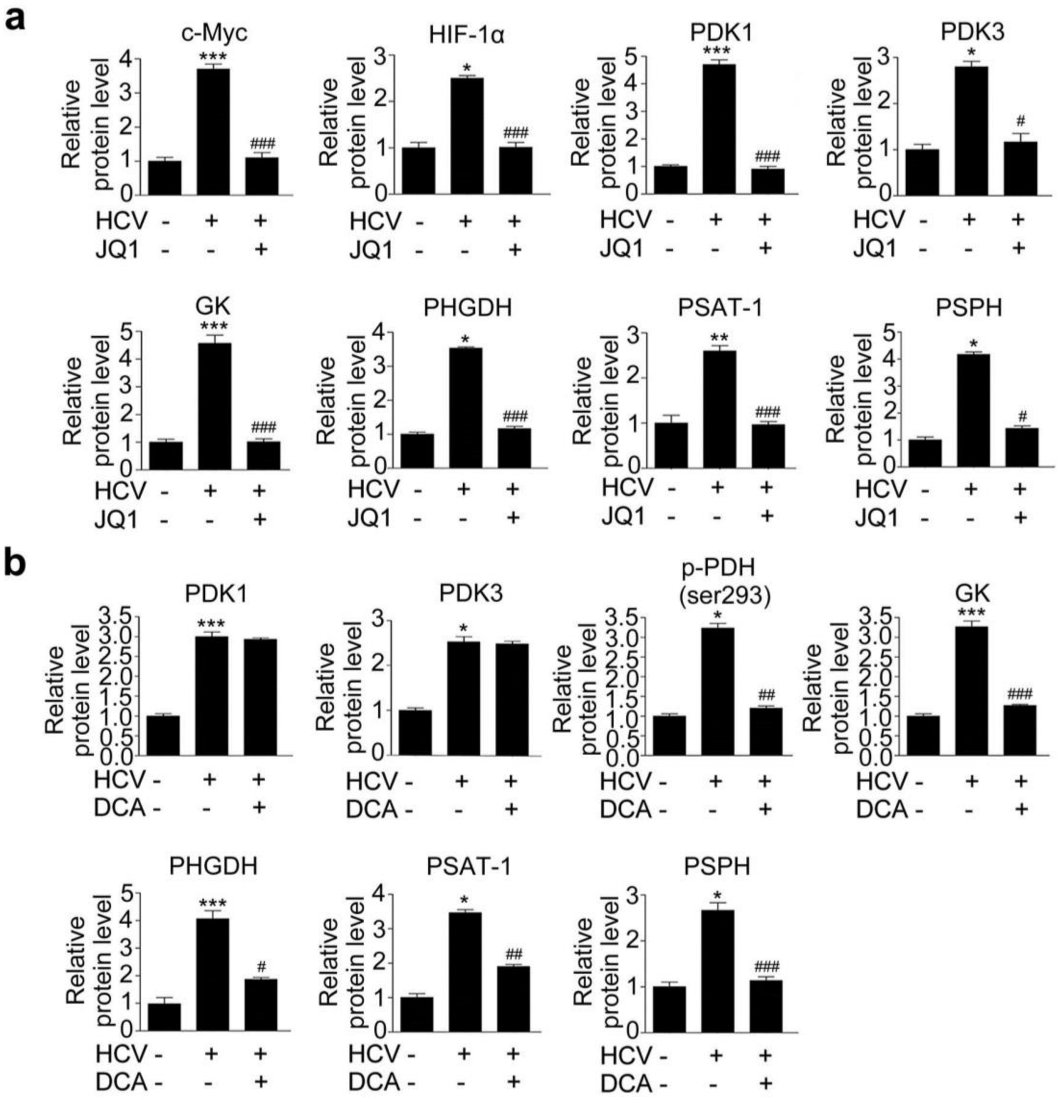

Supplementary Fig. S3. Inhibition of c-Myc and PDKs inhibits the protein levels of enzymes involved in glycolysis and serine biosynthesis in JFH-1-infected Huh-7.5 cells. (a) Relative protein abundance of c-Myc, HIF-1α, PDK1, PDK3, GK, PHGDH, PSAT-1, and PSPH in JFH-1-infected cells treated with or without JQ1 (500 nM, 3 days). Quantitation of immunoblot band intensities are expressed as means ± SEM of three independent experiments. β-tubulin was analyzed as an internal control. (b) Relative phospho-PDH Ser293 levels and protein abundance of GK, PHGDH, PSAT-1, and PSPH in JFH-1-infected cells treated with or without

DCA. Quantitation of immunoblot band intensities are expressed as means ± SEM of three independent experiments. β-tubulin was analyzed as an internal control. $^{*}P$ <0.01, $^{**}P$ <0.05, $^{***}P$ <0.001 compared with uninfected control cells; $^{\#}P$ <0.01, $^{\#\#}P$ < 0.05, $^{\#\#\#}P$ < 0.001 compared with HCV infection alone.

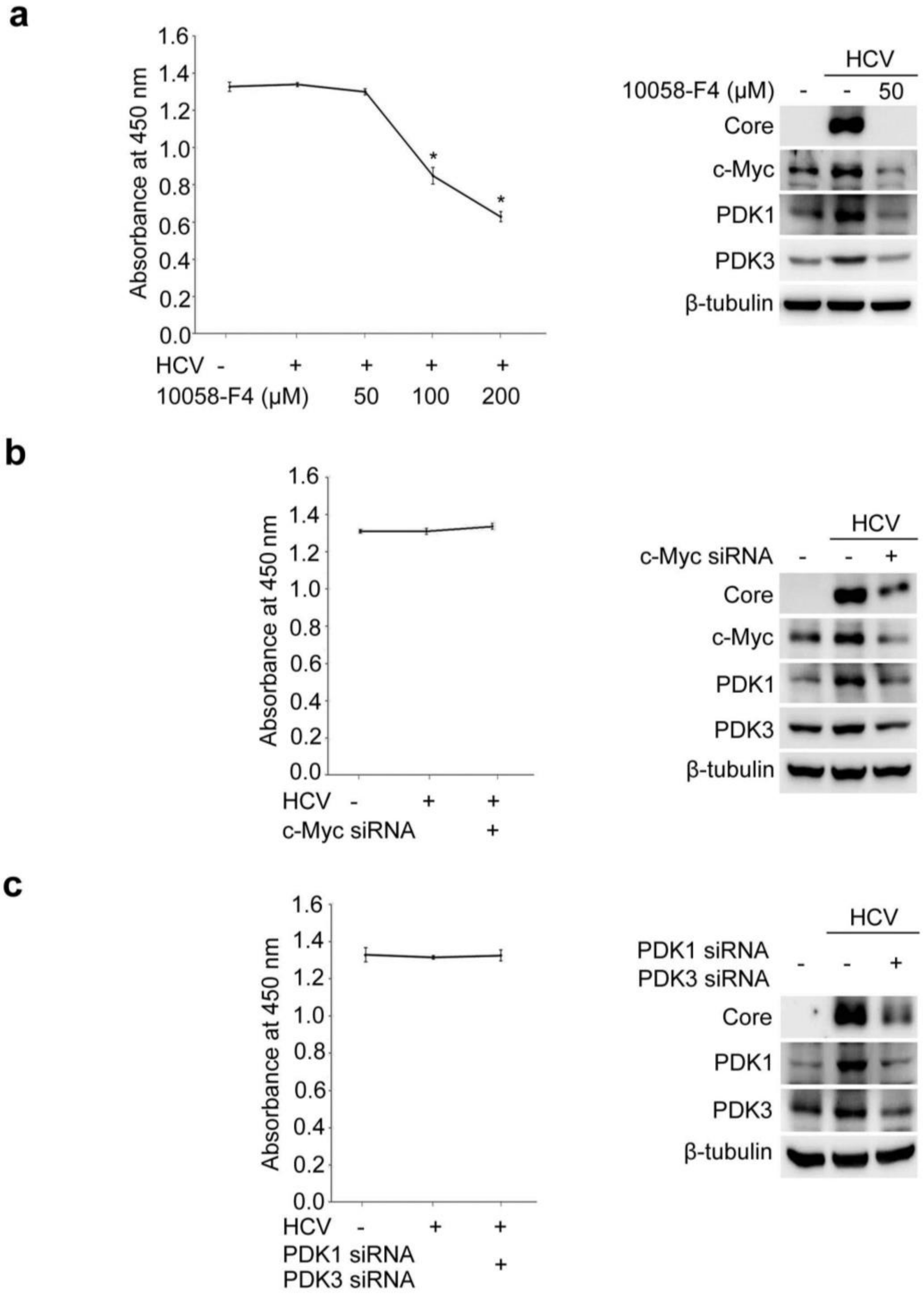

Supplementary Fig. S4. Treatment with the c-Myc inhibitor 10058-F4 and genetic silencing of either c-Myc or PDK inhibit HCV replication in JFH-1-infected Huh7.5 cells. (a) Cytotoxic effects of different doses of a c-Myc inhibitor, 10058-F4, as measured by CCK-8

colorimetry (treatment time, 3 days) (left panel). Data are expressed as the mean ± SEM of three independent measurements. $^{*}P < 0.001$ compared with untreated control cells. Representative immunoblots showing core, c-Myc, PDK1, and PDK3 protein abundance in JFH-1-infected cells treated with 1058-F4 (right panel). (b) Cytotoxicity of c-Myc siRNA as analyzed by CCK-8 colorimetry of JFH-1-infected cells transfected in the presence/absence of c-Myc siRNA (100 nM for 3 days) (left panel). Data are expressed as the mean ± SEM of three independent measurements. Representative immunoblot showing core, c-Myc, PDK1, and PDK3 protein abundance in JFH-1-infected cells transfected with control (scrambled) siRNA or c-Myc siRNA (right panel). (c) Cytotoxicity of PDK1 and PDK3 siRNA in JFH-1-infected cells transfected with control (scrambled) siRNA or PDK1 and PDK3 siRNAs (100 nM for 3 days) (left panel). Data are expressed as the mean ± SEM of three independent measurements. Representative immunoblot showing core, PDK1, and PDK3 protein abundance in JFH-1-infected cells transfected with PDK1 and PDK3 siRNAs (right panel).

Supplementary Material and Methods

Cell culture and reagents

Huh-7.5, a human hepatoma cell line, was obtained from Apath (Brooklyn, NY, USA). Huh-7.5 cells were cultured in an atmosphere containing 5% CO_2 at 37°C in Dulbecco's modified Eagle's medium (DMEM) (1 g/L glucose, 4 mM L-glutamine) containing 10% FBS, 25 mM HEPES, and 1% of 10,000 Units/mL penicillin/10,000 μg/mL streptomycin.. Anti-core antibody was purchased from Thermo Scientific (Waltham, MA, USA). Anti-GK and anti-PHGDH antibodies were purchased from Santa Cruz Biotechnology (Dallas, TX, USA). Anti-PSAT-1 antibody was purchased from Abnova (Taipei, Taiwan). Anti-HIF-1α antibody was purchased from Novus Biologicals (Littleton, CO, USA). Anti-c-Myc antibody was purchased from Abcam (Cambridge, MA, USA). The anti-phospho-PDH Ser 293 antibody was purchased from Millipore (Bedford, MA, USA). A β-tubulin antibody was purchased from Abcam (Cambridge, UK), and the anti-PSPH antibody and sodium DCA, JQ1, 10058-F4 (a c-Myc inhibitor), and ribavirin were purchased from Sigma (St. Louis, MO, USA). Recombinant IFN-α 2b (Intron A) was obtained from Merck Sharp &Dohme (MSD) (Kenilworth, NJ, USA).

Immunofluorescence

To confirm infection, immunofluorescence analysis was performed. Huh-7.5 cells were seeded on slides and infected with HCV. The cells were washed with ice-cold phosphate-buffered saline (PBS), fixed with 4% paraformaldehyde for 10 min at room temperature, and then permeabilized with 0.1 M glycine and 0.1% Triton X-100 for 15 min. Fixed and permeabilized cells were

incubated with anti-core (1:100) antibody overnight at 4℃. After washing, cells were incubated with Alexa Fluor 488-conjugated secondary antibody (Invitrogen, Karlsruhe, Germany) at room temperature for 3 h. DNA was stained with Hoechst 33342 (Pierce Chemical Company, Rockford, IL, USA). Cells were visualized by Nikonfluorescence microscope (Eclipse-80i, Nikon Corporation, Tokyo, Japan).

Immunoblot analysis

Cells (either from cultures or human tissue) were washed twice with PBS and suspended in RIPA buffer. The cells were then lysed on ice for 30 min, and the cell lysate was collected by centrifugation at 15000 × *g* for 10 min and recovery of the resultant supernatant. Protein concentrations were determined using a protein assay kit (Bio-Rad, Hercules, CA, USA). Cell lysates were subjected to sodium dodecyl sulfate polyacrylamide gel electrophoresis (SDS-PAGE), and the resolved proteins were electro-transferred onto a PVDF membrane (Millipore). The membrane was blocked with 5% skim milk in TBS containing 0.1% Tween 20 (TBST) for 1 h, and then incubated with anti-core (1:1000), anti-c-Myc (1:1000), anti-HIF-1α (1:1000), anti-PDK1(1:1000), anti-PDK3(1:1000), anti-phospho-PDH Ser293(1:1000), anti-GK (1:1000), anti-PHGDH (1:1000), anti-PSAT-1 (1:1000),or anti-PSPH(1:1000) antibody at 4℃ with gentle shaking overnight. For the anti-core and anti-β-tubulin antibodies, incubation was for 2 h at room temperature. The membrane was then washed three times in TBST for 10 min. The antibodies were detected using a horseradish peroxidase-linked secondary antibody (Santa Cruz Biotechnology) and the ECL Western Blotting Detection System (Amersham, Buckinghamshire, UK). Each membrane was re-blotted with anti-β-tubulin antibody to verify equal loading of protein in each lane.

Determination of the EC_{50} of DCA required for the inhibition of HCV replication

To measure EC_{50} value, JFH-1-infected Huh7.5 cells were treated with DCA (1 mM to 12 mM). Quantitative real-time RT-PCR was performed using the TaqMan PCR Master Mix Kit on a StepOnePlus™ Real-Time PCR System. The data was calculated using Graphpad prism (San Diego, CA,USA .)

Quantitative Real-time RT-PCR

Total RNA was isolated using the RNeasy Mini Kit (Qiagen, Valencia, CA, USA). cDNAs were synthesized using Thermo Scientific Maxima First strand cDNA Synthesis Kit for RT-qPCR (Thermo scientific).Quantitative real-time RT-PCR was performed using the TaqMan PCR Master Mix Kit (Applied Biosystems, Warrington, UK) on a StepOnePlus™ Real-Time PCR System (Applied Biosystems). Amplifications were conducted in triplicate using specific primers and 6-carboxyfluorescein (FAM)- and tetrachloro-6-carboxyfluorescein (TAMRA)-labeled probes (Applied Biosystems): HCV TaqMan probe, 5'-FAM-CTG CGG AAC CGG TGA GTA CAC-TAMRA-3'; HCV sense primer, CGG GAG AGC CAT AGT GG; HCV antisense primer, AGT ACC ACA AGG CCT TTC G. Reaction conditions were as follows: 2 min at 50℃, 10 min at 95℃, 40 cycles of 15 sec at 95℃, and 1 min at 60℃. At the termination of each PCR run, the data were analyzed by the automated system, and amplification plots were generated. Standard HCV RNA **(**10^2 to 10^8 copies/μg) was used for comparison.

SYBR Real-time RT-PCR

Quantitative real-time RT-PCR was performed using the SYBR Green PCR Master Mix Kit (Applied Biosystems) on a StepOnePlus™ Real-Time PCR System (Applied Biosystems).

Thermal cycling conditions were as follows: 95℃ for 10 min, followed by 40 cycles of 95℃ for 15 s, and 60℃ for 1 min. The sequences of the primers, which were designed using the AB StepOne software (v2.1) based on the relevant sequences from GenBank, were as follows: human Myc (sense, 5'-CGA GGA GGA GAA CTT CTA CCA GC -3'; antisense, 5'-CGA GAA GCC GCT CCA CAT ACA CTC C -3'), human HIF-1α (sense, 5'- ACA GTA TTC CAG CAG ACT CAA -3'; antisense, 5'- CCT ACT GCT TGA AAA AGT GAA -3'), human PDK1 (sense, 5'-AAG CAG TTC CTG GAC TTC GG-3'; antisense, 5'-ACA TTC TGG CTG GTG ACA GG-3'), , human PDK3 (sense, 5'-CTT AAC CGC CCT TCA GTG GG-3'; antisense, 5'-ATG CGG AAA GAG ATG CGG TT-3'), human GK (sense, 5'-TGA CCG GCA CTG CTG AGA T-3'; antisense, 5'-CTT GTC CAG GAA GTC GGA GAT G-3'), human PHGDH (sense, 5'-TGA CAA CAC CTT TGC CCA GT-3'; antisense, 5'-AGC TTC TGC CAG ACC AAT CC-3'), human PSAT-1 (sense, 5'-CAT CCG GGC CTC TCT GTA TA-3'; antisense, 5'-AGG CGG CCA GCT TCT GA-3'), human PSPH (sense, 5'-TGT CAG AAA TGA CAC GGC GA-3'; antisense, 5'-GTA GGC GAC TTA CCA GCT CC-3'), and human GAPDH (sense, 5'-GGA GCC AAA AGG GTC ATC AT-3'; antisense, 5'-GTG ATG GCA TGG ACT GTG GT-3'). Reaction specificity was confirmed by melting curve analysis. The housekeeping gene GAPDH was used as an internal standard.
